# Synthesis of the Supramolecular Structure of Vanadium Pentoxide Nanoparticles with Native and Modified β-Cyclodextrins for Antimicrobial Performance

**DOI:** 10.3390/bioengineering12101010

**Published:** 2025-09-23

**Authors:** Rajaram Rajamohan, Kanagaraj Thamaraiselvi, Chaitany Jayprakash Raorane, Kuppusamy Murugavel, Chandramohan Govindasamy, Seong-Cheol Kim, Seho Sun

**Affiliations:** 1 School of Chemical Engineering, Yeungnam University, Gyeongsan 38541, Republic of Korea; 2 PG and Research Department of Chemistry, Government Arts College, Chidambaram 608102, Tamil Nadu, India; 3 Department of Community Health Sciences, College of Applied Medical Sciences, King Saud University, P.O. Box 10219, Riyadh 11433, Saudi Arabia

**Keywords:** V_2_O_5_ nanoparticles, Beta-cyclodextrins, SEM images, NMR analysis, antimicrobial activity

## Abstract

Cyclodextrins in metal oxide nanoparticles (NPs) serve as stabilizing, dispersing, and functionalizing agents that enhance antimicrobial performance through better nanoparticle stability, synergistic action, and potential controlled release mechanisms, making them ideal for advanced biomedical and environmental antimicrobial applications. In this study, NPs of vanadium pentoxide (V_2_O_5_) were obtained by the precipitation method, and, following a supramolecular assembly, were synthesized using the impregnation method via addition of β-cyclodextrin (BCD) and its derivatives, such as hydroxypropyl-β-cyclodextrin (HCD) and methyl-β-cyclodextrin (MCD). The formation of the V_2_O_5_:CDs was driven by non-covalent host–guest interactions, leading to a stable supramolecular structure with enhanced physicochemical properties. Morphological analysis using scanning electron microscopy (SEM) revealed uniformly distributed V_2_O_5_ NPs within the CD matrix. Structural characterization was further supported by proton nuclear magnetic resonance (NMR) spectroscopy, which confirmed the inclusion interactions between V_2_O_5_ and CDs. The synthesized NPs demonstrated significant antimicrobial activity against Gram-positive and fungal strains, indicating a synergistic enhancement in bioactivity due to the supramolecular architecture. This work highlights the potential of CD-assisted V_2_O_5_ NPs as promising antimicrobial agents for biomedical and environmental applications.

## 1. Introduction

Nanoparticles (NPs) exhibiting antimicrobial properties have gained significant attention in the field of active food packaging due to their ability to enhance the shelf life and safety of perishable products [1,2]. When incorporated into composite functional films, these nanoparticles serve as active agents that inhibit the growth of spoilage microorganisms, including bacteria, yeasts, and molds, on the surface of food products. Their presence helps to reduce microbial contamination, thereby maintaining the quality, safety, and freshness of the packaged food throughout storage and distribution. This innovative approach not only extends the product’s shelf life but also minimizes the reliance on chemical preservatives, aligning with current consumer preferences for cleaner and more sustainable packaging solutions [3].

Vanadium pentoxide nanoparticles (V_2_O_5_ NPs) have attracted significant attention as potent antimicrobial agents due to their unique physicochemical properties, such as high surface area, strong oxidative potential, and versatile redox behavior. These attributes enable V_2_O_5_ NPs to exhibit broad-spectrum antimicrobial activity against a wide range of pathogenic microorganisms, including Gram-positive and Gram-negative bacteria, fungi, and other microbial species [4,5,6]. The primary mechanism of their antimicrobial action involves the catalytic generation of reactive oxygen species (ROS), including hydroxyl radicals (•OH), superoxide anions (O_2_^−^), and hydrogen peroxide (H_2_O_2_). These ROS cause oxidative damage to vital cellular components such as lipids, proteins, and nucleic acids, ultimately compromising microbial cell integrity and leading to cell death [7]. Moreover, V_2_O_5_ NPs can directly interact with microbial membranes, increasing permeability, causing structural disruption, and leading to the leakage of intracellular contents, an effect particularly pronounced in Gram-negative bacteria due to their thinner peptidoglycan layers [8]. The internalization of NPs can also lead to direct interactions with microbial DNA and proteins, disrupting essential processes such as DNA replication and protein synthesis, thereby inhibiting microbial growth and proliferation [9]. Incorporating V_2_O_5_ NPs into biopolymer or polymer-based matrices for food packaging applications further enhances the antimicrobial functionality of the packaging material. Notably, this antimicrobial enhancement does not compromise the mechanical strength, thermal stability, or barrier properties of the packaging film. Consequently, V_2_O_5_-loaded films offer a promising solution for active food packaging systems designed to improve food safety and extend shelf life [10].

However, despite their potential, not only V_2_O_5_ NPs but also the other metal oxide NPs face several limitations related to stability, dispersion, safety, and sustained functionality [11,12,13]. These NPs tend to agglomerate in aqueous and polymer matrices due to their high surface energy, which reduces their effective surface area and antimicrobial performance [14]. Moreover, for effective antimicrobial systems, controlled and prolonged release of the active agent is essential to maintain long-term efficacy [14]. Sustaining the antimicrobial effect over time remains a challenge, particularly in biomedical and food-related applications.

To overcome these limitations, cyclodextrins (CDs) have been employed to stabilize and modulate the release behavior of V_2_O_5_ NPs. CDs are cyclic oligosaccharides composed of α-(1,4)-linked glucopyranose units. The three naturally occurring types, alpha-CD (α-CD), beta-CD (β-CD), and gamma-CD (γ-CD), contain six, seven, and eight glucose units, respectively. Their truncated cone-shaped structure allows them to form stable inclusion complexes with various guest molecules [15,16]. While CDs themselves lack intrinsic antimicrobial activity, they are widely used in food packaging to encapsulate and enable the sustained release of antimicrobial and antioxidant agents [17,18].

Recently, several studies have reported the functionalization of metal oxide NPs with BCD to improve their stability, surface modification, and biological performance. For example, BCD/Fe_2_O_3_ nanostructures have demonstrated excellent antibacterial efficiency through green synthesis strategies [19,20]. Similarly, BCD–TiO_2_ nanocomposites loaded with sorbic and benzoic acids showed improved antimicrobial activity [21], while Aloe vera-mediated ZnO–BCD nanocomposites exhibited enhanced biocompatibility and antimicrobial potential [22]. Beyond antimicrobial applications, BCD-stabilized cupric oxide NPs have also been explored for thermal therapy, showing promising in vitro anticancer activity against lung tumors [23]. Accordingly, the present study aims to develop a CD-based V_2_O_5_ NP system designed for enhanced stability and controlled, sustained antimicrobial release. Both natural and modified CDs are utilized to synthesize stabilized V_2_O_5_ NPs, allowing for a comparative evaluation of their antimicrobial performance.

## 2. Results and Discussion

### 2.1. Characterization of V_2_O_5_ NPs, Including Loaded with CDs

#### 2.1.1. Powder XRD Analysis

**Figure 1:**
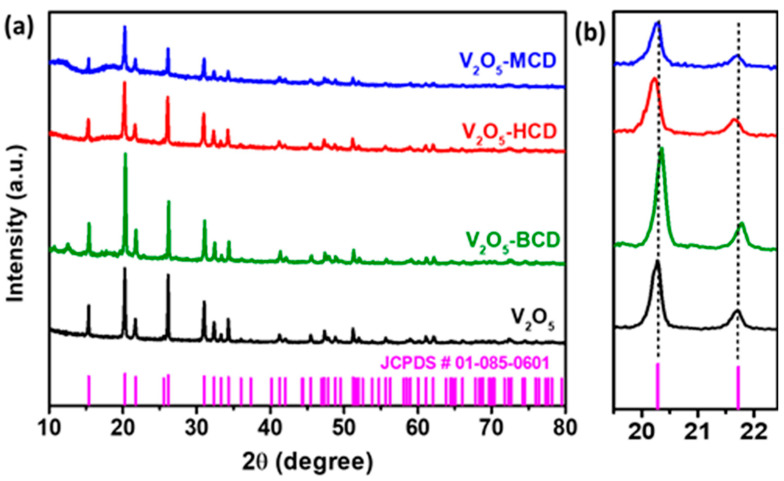
XRD patterns of as-synthesized V_2_O_5_ and V_2_O_5_ loaded with CDs (**a**), and expanded view of peak shifting (**b**).

Figure 1 presents the XRD patterns of the as-synthesized V_2_O_5_ and V_2_O_5_:CD samples. The XRD profile of pure V_2_O_5_ exhibits distinct diffraction peaks at 2θ values of 15.37°, 20.29°, 21.72°, 25.56°, 26.15°, 31.03°, 32.38°, 33.33°, 34.30°, 41.26°, 45.46°, 47.31°, 51.22°, 51.98°, 55.65°, 58.49°, 58.99°, 61.11°, 62.07°, 66.03°, 72.28°, 74.48°, and 78.17°, which correspond to the (020), (001), (011), (021), (110), (031), (101), (111), (130), (002), (141), (060), (200), (061), (201), (161), (142), (231), (170), (171), (260), (133), and (143) crystal planes, respectively. These reflections are in good agreement with the standard orthorhombic phase of V_2_O_5_, as referenced by JCPDS card no. 01-085-0601. The identified crystal structure belongs to the orthorhombic system with a Pmmn space group, indicating the successful formation of phase-pure V_2_O_5_. Upon incorporation of CDs into the V_2_O_5_ matrix, the XRD patterns of the resulting V_2_O_5_:CD retained all the characteristic peaks of V_2_O_5_, suggesting that the primary crystal structure of V_2_O_5_ remained intact. However, slight shifts in peak positions along with variations in intensity were observed, indicating that the presence of CD molecules had induced minor structural modifications without forming any new crystalline phases. This confirms that the CDs interacted with V_2_O_5_ primarily through surface adsorption or physical encapsulation rather than chemical transformation [24,25,26]. A magnified view of the (001) and (021) reflections is shown in Figure 1b to highlight these subtle changes. Among the three CD-modified samples, BCD incorporation resulted in an increased intensity of the diffraction peaks, indicating enhanced crystallinity of the V_2_O_5_ phase. In contrast, V_2_O_5_:MCD and V_2_O_5_:HCD exhibited peak broadening and a noticeable reduction in intensity, which were indicative of reduced crystallite size and partial lattice distortion.

These changes can be attributed to the influence of CD size and functional groups on the nucleation and growth dynamics of V_2_O_5_ NPs. The decrease in peak intensity and broadening in V_2_O_5_:MCD and V_2_O_5_:HCD suggest the formation of smaller and more disordered nanocrystals due to interference in the growth process. Additionally, a slight shift of peaks toward lower 2θ values was observed, particularly in V_2_O_5_:MCD and V_2_O_5_:HCD. This shift was most likely due to compressive strain introduced into the V_2_O_5_ lattice by the interaction with bulky CD moieties, which may have distorted the local lattice environment [27].

#### 2.1.2. FE-SEM Analysis

**Figure 2:**
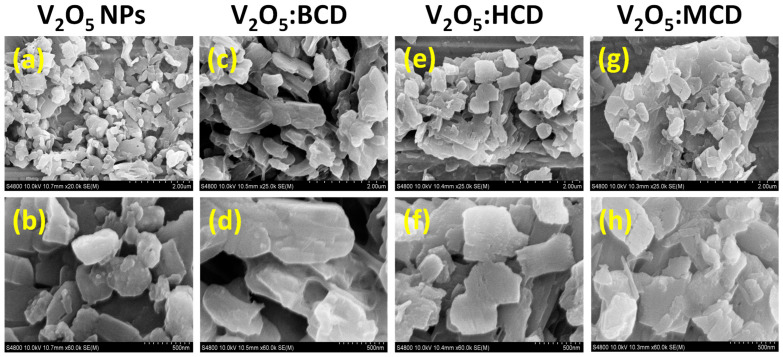
FE-SEM images of V_2_O_5_ (**a**,**b**), V_2_O_5_:BCD (**c**,**d**), V_2_O_5_:HCD (**e**,**f**), and V_2_O_5_:MCD (**g**,**h**).

The surface morphology and microstructural characteristics of the as-synthesized V_2_O_5_ and V_2_O_5_:CDs were analyzed using FE-SEM, as shown in Figure 2. SEM images were captured at two different magnifications (2 µm and 500 nm) to investigate the particle shape, size, and agglomeration behavior in greater detail. Figure 2a,b depicts the morphology of pristine V_2_O_5_, revealing a densely packed structure with significant nanoparticle agglomeration. The NPs appear irregular and tend to cluster, which was commonly observed due to the high surface energy and interparticle interactions inherent to nanomaterials. Incorporation of CDs into the V_2_O_5_ matrix led to noticeable changes in surface characteristics without altering the fundamental morphology of V_2_O_5_. V_2_O_5_:BCD, shown in Figure 2c,d, retained the intrinsic morphology of V_2_O_5_ but exhibited a more dispersed arrangement, with a significant reduction in agglomeration. This suggests that BCD molecules were effectively adsorbed on the surface of V_2_O_5_ NPs, acting as a steric stabilizer that prevented particle–particle aggregation [28]. The slight increase in particle size observed in the loaded material may be attributed to the coating of BCD on the NP surfaces, forming a protective shell around each particle. In the case of V_2_O_5_:HCD, shown in Figure 2e,f, a moderate reduction in agglomeration was observed when compared to pristine V_2_O_5_, though not as pronounced as in the BCD. The particles appeared more loosely packed, indicating partial stabilization by HCD. This intermediate behavior may be due to differences in the interaction strength or spatial arrangement between HCD and the surface of V_2_O_5_ NPs.

Interestingly, the V_2_O_5_:MCD, presented in Figure 2g,h, exhibited a higher degree of agglomeration than the pure V_2_O_5_. The presence of MCD appears to promote clustering rather than dispersion, possibly due to weaker surface interaction or an inadequate steric hindrance effect. Similarly, MCD has a methylation surface that greatly inhibits the capacity to make hydrogen bonds with V_2_O_5_ NPs, which leads to more agglomeration in SEM investigations than BCD and HCD. A weaker, less effective protective shell was produced by MCD’s fewer hydroxyl groups than by BCD and HCD, which have more hydroxyl groups for efficient capping. This makes it easier for NPs to assemble since there is less steric impediment between them. Also, effective capping reduces agglomeration in V_2_O_5_:BCD, resulting in a uniform elemental distribution and great matrix stability. In contrast, a clustered element distribution and enhanced agglomeration were observed in V_2_O_5_:MCD due to less efficient capping. For applications that require evenly distributed NPs, the V_2_O_5_:MCD was less appropriate due to its decreased stability. This suggests that among the three CDs used, BCD was the most effective in mitigating particle agglomeration, followed by HCD, while MCD may be less suitable for stabilizing V_2_O_5_ NPs under the given synthesis conditions.

**Figure 3:**
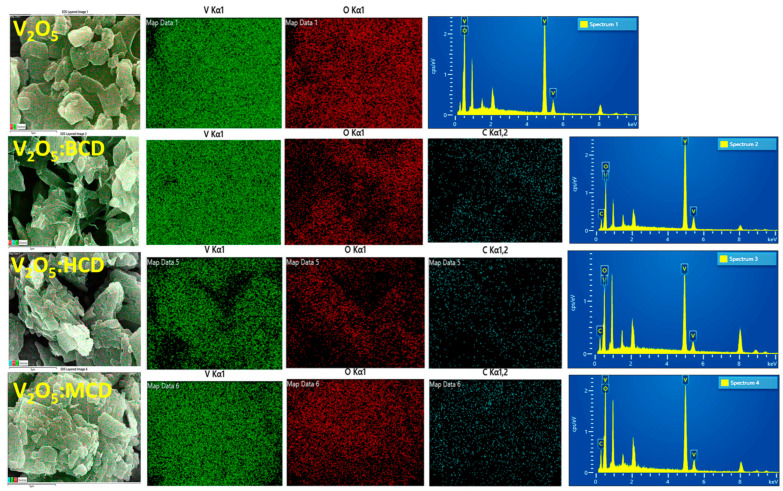
EDAX outputs for V_2_O_5_, V_2_O_5_:BCD, V_2_O_5_:HCD, and V_2_O_5_:MCD.

To complement the morphological analysis, elemental mapping and EDAX spectroscopy were performed to confirm the elemental composition of the samples (Figure 3). For the pristine V_2_O_5_ sample, elemental mapping revealed a uniform distribution of vanadium (V) and oxygen (O) throughout the sample, consistent with the expected stoichiometry of V_2_O_5_. The corresponding EDAX spectrum also confirmed the exclusive presence of V and O elements. In V_2_O_5_:CD, the elemental mapping indicated homogeneous distribution of vanadium, oxygen, and carbon (C), with the presence of carbon attributed to the organic CD molecules. The EDAX spectra for V_2_O_5_:BCD, V_2_O_5_:HCD, and V_2_O_5_:MCD further validated this, showing clear peaks corresponding to V, O, and C, thereby confirming successful incorporation of the cyclodextrin moieties into the V_2_O_5_ NPs.

#### 2.1.3. FT-IR Spectral Analysis

**Figure 4:**
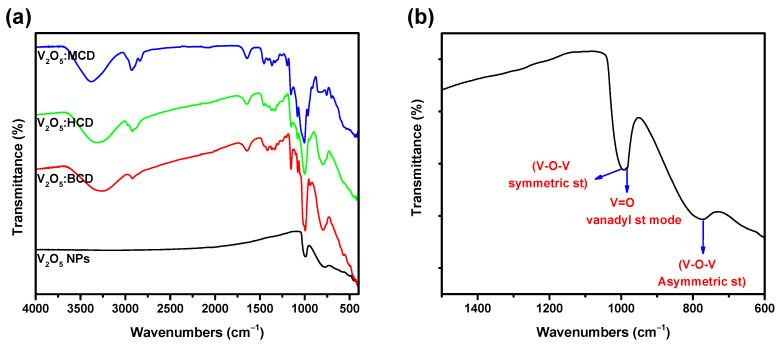
FT-IR spectra of V_2_O_5_ NPs and their BCD−loaded products (**a**), and expanded spectra of V_2_O_5_ NPs (**b**).

The FT-IR spectra of both unloaded and CD-loaded V_2_O_5_ NPs are shown in Figure 4a. For the pure V_2_O_5_ NPs (Figure 4b), three predominant vibrational modes were clearly distinguished: the V–O–V symmetric stretching at ~1080 cm^−1^, the V–O–V asymmetric stretching at ~795 cm^−1^, and the terminal V=O vanadyl stretching mode at ~1004 cm^−1^. These characteristic absorptions were in good agreement with previously reported assignments for orthorhombic V_2_O_5_ and strongly corroborate the crystallographic findings obtained from XRD analysis [29]. The appearance of these well-defined stretching modes confirms the structural integrity and phase purity of the synthesized V_2_O_5_ NPs.

The spectral analysis can be interpreted from two perspectives: the first relates to the vibrational changes associated with the NPs, while the second focuses on the characteristic vibrations of the CDs. Upon incorporation of CDs (BCD, HCD, and MCD), slight but discernible modifications were observed in the V–O vibrational region. In particular, the V=O stretching band at ~1004 cm^−1^ exhibited a marginal shift and intensity variation, suggesting weak coordination or hydrogen bonding interactions between the oxygen atoms of V_2_O_5_ and the hydroxyl functionalities of the CDs. Similarly, the V–O–V stretching modes at 1080 and 795 cm^−1^ showed subtle broadening, which can be attributed to local changes in the vibrational environment caused by supramolecular encapsulation. These spectral perturbations provide additional evidence for the successful integration of V_2_O_5_ within the CD matrix and the establishment of host–guest interactions.

The existence of the −OH stretching group in CD was verified by the spectral bands observed at ~3430 cm^−1^. Upon interaction with a V_2_O_5_, the band shifts to ~3395 cm^−1^, exhibiting increased broadening for all the CD-based products. However, the bands at this range for MCD were slightly less broad than those of other products with BCD and HCD. The spectral feature observed at 2930 cm^−1^ can be attributed to the stretching vibration of the C–H functional group. The minimal shift was observed in this region for all the products. The presence of spectral bands at ~1605 cm^−1^ may be ascribed to the stretching vibration of C═C and −C–O groups and the deformation of the aromatic ring. Obtaining V_2_O_5_ NPs with CDs resulted in splitting the band at 1605 cm^−1^ of CDs into less intense and weaker [19,30]. The above changes confirmed the hydrogen bonding interaction of NPs with other CDs via the O and H at NPs and CDs, respectively. The FT-IR spectra provided clear evidence of the interaction between V_2_O_5_ NPs and CDs. The characteristic broad absorption band of free CDs observed at ~3430 cm^−1^ corresponds to the stretching vibration of the hydroxyl (−OH) groups, which originate from both primary and secondary hydroxyl functionalities on the CD skeleton. Upon hybridization with V_2_O_5_, this band exhibited a noticeable shift to ~3395 cm^−1^, accompanied by increased broadening. This broadening was indicative of stronger hydrogen-bonding interactions between the −OH groups of CDs and oxygen atoms on the surface of V_2_O_5_ NPs. Notably, among the studied systems, MCD displayed a comparatively less broadened feature in this region than BCD and HCD, suggesting that the substitution pattern of MCD may sterically hinder the extent of hydrogen bonding with V_2_O_5_. The absorption band observed at ~2930 cm^−1^ was attributed to the C–H stretching vibrations of the glucopyranose units of CDs. Only minimal shifts were detected in this region across all V_2_O_5_:CDs, implying that the hydrophobic C–H environments were less perturbed by the incorporation of NPs. Another important spectral feature was the band at ~1605 cm^−1^, which arises from a combination of C═C stretching vibrations, −C–O stretching, and deformation modes of the CD aromatic skeleton. In pristine CDs, this band appeared as a strong, well-defined signal; however, upon hybridization with V_2_O_5_, the band underwent splitting into weaker and less intense components [19,30]. This splitting was a strong indication of the disruption of the original CD vibrational environment and the establishment of host–guest interactions between V_2_O_5_ NPs and CDs. Taken together, these spectral changes, specifically the downshift and broadening of the hydroxyl stretching band and the splitting of the ~1605 cm^−1^ band, confirm that hydrogen bonding plays a dominant role in stabilizing the V_2_O_5_:CD nanohybrids. The interactions were most likely mediated through the hydroxyl protons of CDs engaging with oxygen atoms on the V_2_O_5_ surface, creating a supramolecular network that enhances both stability and dispersion of the NPs within the CD matrix.

#### 2.1.4. ^1^H NMR Spectral Analysis

##### ^1^H NMR Spectral Analysis of BCD

The ^1^H NMR spectrum of BCD is presented in Figure S1. The spectrum displays characteristic signals corresponding to the protons of the BCD, consistent with its toroidal cyclic oligosaccharide structure. Multiplet resonances observed in the upfield region at 3.30, 3.56, and 3.63 ppm were assigned to the methine (–CH) and methylene (–CH_2_) protons of the glucose units forming the BCD ring. These signals reflect the influence of intramolecular hydrogen bonding and the spatial orientation within the macrocyclic cavity. A well-defined triplet at 4.45 ppm (J ≈ 5.4 Hz) was attributed to the hydroxyl proton of the hydroxy-methylene group at the C-5 position of the glucopyranose unit. This resonance supports the presence of intramolecular hydrogen bonding involving the secondary hydroxyl groups. Additionally, two downfield doublets at 5.73 and 5.67 ppm, with coupling constants of ~3 Hz and ~7.2 Hz, were assigned to the equatorial hydroxyl protons at the C-2 and C-3 positions, respectively. Their downfield chemical shifts suggest deshielding effects arising from hydrogen bonding and ring strain within the BCD structure. A distinct signal at 4.82 ppm, appearing as a singlet with a small coupling constant (J = 3.6 Hz), corresponded to the anomeric proton (H-1) of BCD. This resonance is characteristic of the hydrogen attached to the C-1 position in the α-1,4 glycosidic linkage and confirms the integrity of the glycosidic bonds within the cyclic structure. Furthermore, a broad multiplet spanning 3.59–3.68 ppm was assigned to the methylene protons at the C-5 position, reflecting their sensitivity to the surrounding chemical environment inside the BCD cavity. A comprehensive summary of the proton assignments and corresponding chemical shifts is provided in Table S1, further validating the structural integrity and purity of the BCD employed in this study [31].

##### ^1^H NMR Spectral Analysis of V_2_O_5_-Loaded BCD

**Figure 5:**
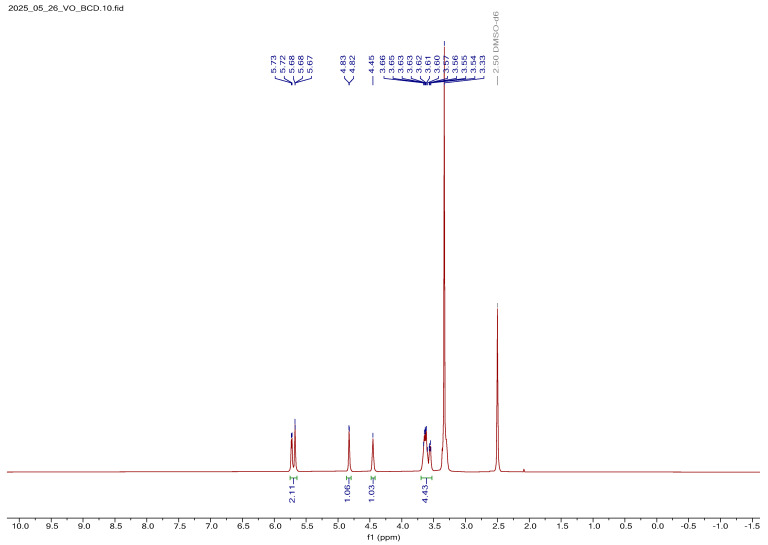
Proton NMR spectra of V_2_O_5_:BCD.

The ^1^H NMR spectrum of V_2_O_5_:BCD is shown in Figure 5. Upon comparison with the spectrum of free BCD, noticeable changes were observed, indicating successful interaction between the BCD host and V_2_O_5_ NPs. Specifically, the equatorial hydroxyl protons located at the C-2 and C-4 positions, along with the hydroxyl proton of the methylene group at the C-5 position, experienced a slight upfield shift of approximately 0.01 ppm. This minor shielding effect suggests weak electronic interactions or hydrogen bonding between these hydroxyl groups and the loaded V_2_O_5_ species. Additionally, the characteristic sharp proton signals seen in the spectrum of free BCD were noticeably broadened in the V_2_O_5_-loaded BCD in the form of a complex. This line broadening was attributed to the restricted molecular motion and altered local environment caused by the inclusion or surface adsorption of V_2_O_5_ within or onto the BCD matrix. The broadening effect further supports the presence of V_2_O_5_ and indicates partial complexation or physical interaction between the guest and host molecules.

Interestingly, despite these changes, the overall chemical shifts of other BCD protons remained largely unaffected, implying that the core cyclic structure of BCD was preserved upon V_2_O_5_ loading. However, a notable increase in the coupling constants was observed, approximately double the values seen in free BCD, indicating increased rigidity or conformational restriction of the glucose rings due to the incorporation of V_2_O_5_. Based on these spectral changes, it can be concluded that V_2_O_5_ was associated with the BCD moiety, likely via surface adsorption or weak inclusion interactions, rather than covalent bonding. This conclusion was further corroborated by SEM analysis, which visually confirms the presence of V_2_O_5_ NPs distributed alongside the BCD matrix. A detailed summary of the proton chemical shifts for V_2_O_5_:BCD is provided in Table S1.

##### ^1^H NMR Spectral Analysis of HCD

The ^1^H NMR spectrum of HCD is shown in Figure S2. In addition to the characteristic proton signals of the native BCD backbone, several new resonances were observed, confirming the successful substitution of hydroxypropyl groups onto the CD cavity. A notable upfield multiplet at 1.03 ppm was assigned to the methyl (–CH_3_) protons of the hydroxypropyl side chains. This signal serves as a distinct marker of the introduced propyl groups and is consistent with previously reported values for similar substitutions. Multiplets detected in the regions of 3.56–3.59 ppm and 4.46–4.50 ppm were attributed to the methylene (–CH_2_–) and methine (–CH–) protons of the hydroxypropyl substituents. The influence of adjacent hydroxyl and ether functionalities likely contributed to the slight deshielding and signal broadening observed in these regions. A well-defined multiplet at 5.74 ppm corresponded to the hydroxyl proton bound to the secondary carbon of the hydroxypropyl chain. Its downfield shift reflects deshielding effects arising from hydrogen bonding and the proximity of electron-withdrawing oxygen atoms. Furthermore, several broad resonances within the 4.80–5.88 ppm range were assigned to overlapping contributions from the anomeric (H-1) and hydroxyl protons of the glucose units, as well as those introduced through hydroxypropylation. Such overlapping signals are typical of substituted CDs and reflect the structural heterogeneity and variation in substitution degrees. The observed assignments are in good agreement with previously reported chemical shifts for hydroxypropyl-substituted CDs, thereby validating both the structural integrity and the substitution pattern of HCD employed in this study [31].

##### ^1^H NMR Spectral Analysis of V_2_O_5_:HCD

**Figure 6:**
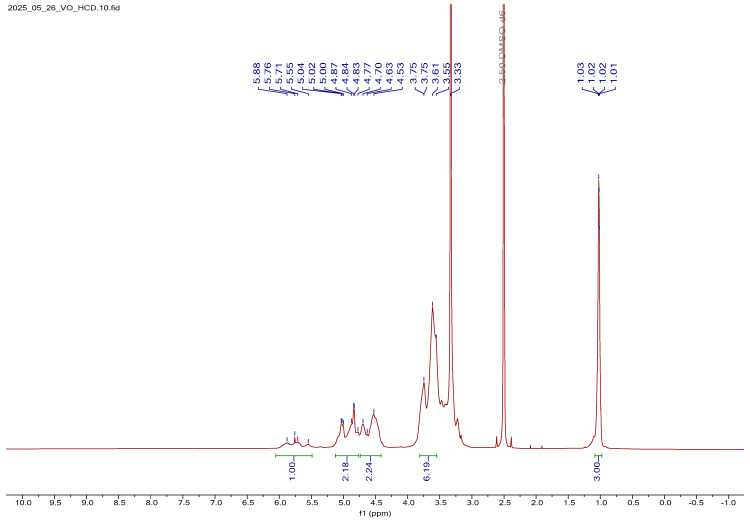
Proton NMR spectra of V_2_O_5_:HCD.

The ^1^H NMR spectrum of V_2_O_5_:HCD is shown in Figure 6. Upon comparison with the spectrum of free HCD, several notable changes in chemical shifts and signal characteristics were observed, providing strong evidence of interaction between the HCD host matrix and V_2_O_5_. In the spectrum, the equatorial hydroxyl protons at the C-2 and C-4 positions of the glucose units showed a downfield shift of approximately 0.3 ppm. This deshielding effect suggests significant electronic perturbation in these regions, likely due to hydrogen bonding interactions between the free hydroxyl groups of HCD and surface oxygen atoms of V_2_O_5_. Such interactions indicate close proximity between V_2_O_5_ and the hydrophilic outer rim of the CD ring. Conversely, the hydroxyl proton of the 2-hydroxypropyl substituent (attached at the C-5 position of the glucose unit) exhibited a slight upfield shift of 0.05 ppm. This minor shielding may arise from reduced hydrogen bonding or conformational changes in the propyl side chain upon complexation with V_2_O_5_. Additionally, a downfield shift of 0.2 ppm was recorded for both the anomeric H-1 proton and the methine proton of the 2-hydroxypropyl group. This shift implies that these protons were situated in a more deshielded environment, possibly due to the spatial rearrangement and altered electron density resulting from the incorporation of V_2_O_5_. Apart from H-2, all other proton signals experienced a slight upfield shift of approximately 0.01 ppm. These subtle shifts, although minimal, indicate small but widespread changes in the chemical environment across the glucose and propyl chains, reflecting a weak yet consistent interaction throughout the host molecule. Moreover, signal broadening was evident throughout the spectrum when compared to the sharp peaks observed in free HCD. This broadening was attributed to the reduced molecular mobility and increased heterogeneity introduced by the presence of V_2_O_5_ NPs, either through surface adsorption or encapsulation within the CD cavity. Despite these spectral modifications, the overall splitting pattern of the protons remained unchanged, indicating that the fundamental structural integrity of the HCD ring system was preserved even after V_2_O_5_ loading. These spectral observations, combined with SEM analysis, confirm the successful incorporation of V_2_O_5_ into the HCD cavity. The SEM results provide complementary morphological evidence supporting the formation of a V_2_O_5_:HCD. A detailed summary of the proton chemical shift values for the V_2_O_5_-loaded HCD (V_2_O_5_:HCD) is provided in Table S2.

##### Analysis of MCD ^1^H NMR Spectrum

The ^1^H NMR spectrum of MCD is presented in Figure S3. Multiplet signals at 5.74 ppm and within the 4.72–5.03 ppm region were assigned to the axial protons of the MCD ring. Resonances corresponding to the methyl protons of methoxy substituents at the C-2 and C-3 positions, as well as the methylene protons of the glucose units, were observed at 3.56 ppm, 3.74 ppm, and within the 4.45–4.54 ppm range, respectively. A distinct singlet at 3.61 ppm was attributed to the methoxy protons of the methyl groups linked to methylene units, partially overlapping with signals from the methylene resonances. The assignment of individual proton chemical shifts was guided by the carbon environment and the nature of the substituents within the CD. A comprehensive summary of these assignments is provided in Table S3, further supporting the structural characterization of MCD [32].

##### ^1^H NMR Spectral Analysis of V_2_O_5_:MCD

**Figure 7:**
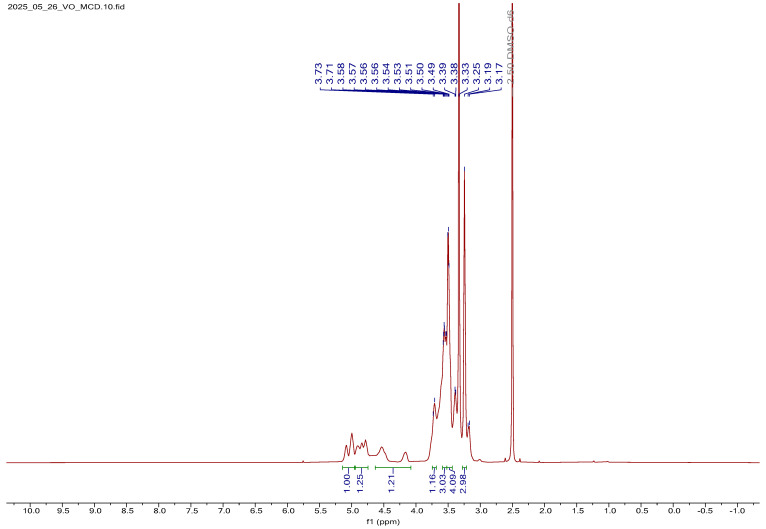
Proton NMR spectra of V_2_O_5_:MCD.

The ^1^H NMR spectrum of V_2_O_5_:MCD is shown in Figure 7. In the spectrum, the methyl protons of the equatorial methoxy groups at C-2 and C-4, as well as the methoxy group attached to the methylene carbon at C-5, exhibited upfield shifts (shielding) ranging from 0.11 to 0.31 ppm. Upon V_2_O_5_ loading onto MCD, the proton signals appeared broadened compared to the sharp peaks observed for the free MCD, indicating interactions between the MCD and V_2_O_5_. The axial protons at H-4 and H-5 remained unaffected, while the H-2 proton showed a downfield shift (deshielding) of 0.15 ppm. All other protons experienced shielding effects ranging from 0.2 to 0.9 ppm, with the methylene protons at C-5 showing the most significant shielding. These observations suggest the successful incorporation of V_2_O_5_ within the MCD. This conclusion was further supported by XRD and SEM analysis, which confirmed the presence of V_2_O_5_ in association with the MCD structure. The detailed proton chemical shift values for V_2_O_5_:MCD are listed in Table S3.

### 2.2. Antifungal and Antibacterial Efficacy of NPs

**Figure 8:**
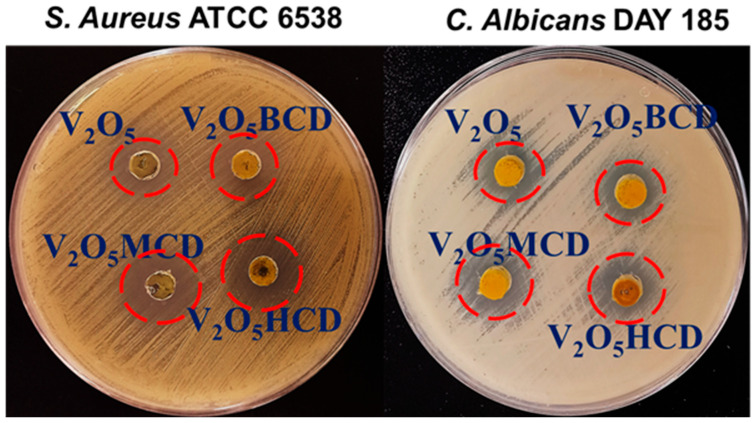
Antibacterial activity of V_2_O_5_, V_2_O_5_:BCD, V_2_O_5_:HCD, and V_2_O_5_:MCD against *S. aureus* ATCC 6538 and *C. albicans* DAY 185. Zones of inhibition were measured in millimeters (excluding the 9 mm well diameter). Data are expressed as mean ± SD from two independent experiments performed in triplicate.

The antifungal susceptibility of *Candida albicans* towards the V_2_O_5_ and V_2_O_5_ loaded with BCD, HCD, and MCD (V_2_O_5_:BCD, V_2_O_5_:HCD, and V_2_O_5_:MCD, respectively) NPs was evaluated using the agar well diffusion assay. Clear and measurable inhibition zones were observed for all tested formulations, confirming their antifungal potential. Among the four NPs, V_2_O_5_:HCD and V_2_O_5_:MCD demonstrated the most pronounced activity, producing more distinct and wider inhibition zones compared to V_2_O_5_ and V_2_O_5_:BCD. Representative inhibition zone images are shown in Figure 8, visually corroborating the enhanced antifungal action of the CD-functionalized systems. Quantitatively, the measured inhibition zone diameters (excluding the 9 mm disc diameter) were 6.2 ± 0.3 mm for V_2_O_5_, 6.4 ± 0.1 mm for V_2_O_5_:BCD, 7.8 ± 0.4 mm for V_2_O_5_:HCD, and 10.2 ± 0.2 mm for V_2_O_5_:MCD, indicating a progressive improvement in antifungal efficacy with host–guest inclusion modifications, particularly in the MCD system (Figure S4). A similar agar well diffusion assay was employed to assess antibacterial efficacy. All four NPs displayed inhibitory activity against *Staphylococcus aureus* (Gram-positive), while *Escherichia coli* (Gram-negative) exhibited complete resistance to all tested formulations (Figure S5). The inhibition zone measurements against *S. aureus* were 7.3 ± 0.2 mm for V_2_O_5_, 8.5 ± 0.3 mm for V_2_O_5_:BCD, 9.3 ± 0.9 mm for V_2_O_5_:HCD, and 11.4 ± 0.9 mm for V_2_O_5_:MCD (Table 1), showing a consistent trend of increased antibacterial activity following CD functionalization, with methylated derivatives again producing the most significant enhancement. The superior antimicrobial activity of V_2_O_5_:MCD likely arises from the combined ROS-mediated effects of V_2_O_5_ and the ability of MCD to enhance NP dispersion, increase bioavailability, and promote stronger interactions with microbial membranes, thereby amplifying synergistic inhibition against *S. aureus* and *C. albicans* [33,34].

The selective antibacterial effect was potent against *S. aureus* but ineffective against *E. coli*, which can be attributed to fundamental structural differences between Gram-positive and Gram-negative bacterial cell envelopes. Gram-positive bacteria, such as *S. aureus*, possess a thick peptidoglycan layer without an outer membrane, facilitating direct adsorption of V_2_O_5_ NPs, potentially mediated by electrostatic interactions. This interaction can lead to membrane destabilization and cell damage, possibly through the generation of reactive oxygen species (ROS) [35,36]. In contrast, Gram-negative bacteria like *E. coli* were protected by an additional outer membrane enriched with lipopolysaccharides (LPS), which can act as a physical and electrostatic barrier, limiting nanoparticle attachment, penetration, and ROS access [37].

The observed antimicrobial selectivity may be attributed to fundamental differences in cell wall architecture between Gram-positive and Gram-negative bacteria. *S. aureus* possesses a thick but permeable peptidoglycan layer that facilitates interaction with V_2_O_5_:CD, whereas *E. coli* has an additional outer membrane rich in lipopolysaccharides (LPS) that acts as a strong permeability barrier, restricting nanoparticle penetration and reducing antimicrobial efficacy. Similar selective activity of nanomaterials toward Gram-positive species has been reported in earlier studies [38,39]. The detailed proposed mechanism is discussed below.

### 2.3. Proposed Antimicrobial Mechanism of V_2_O_5_ NPs

The antimicrobial activity of V_2_O_5_-based NPs was strongly influenced by the structural differences between Gram-positive and Gram-negative bacterial cell envelopes. In Gram-positive bacteria (*S. aureus*), the cell wall was primarily composed of a thick peptidoglycan layer (~20–80 nm) that was directly exposed to the extracellular environment, without the presence of an additional outer membrane. This structural openness facilitates the adsorption of V_2_O_5_ NPs onto the bacterial surface, likely mediated by electrostatic interactions between the negatively charged bacterial cell wall components (e.g., teichoic acids) and the surface charge of the NPs [40,41]. Once adhered, V_2_O_5_ NPs can induce ROS generation, such as superoxide radicals, hydroxyl radicals, and hydrogen peroxide, either via surface redox reactions or through photocatalytic processes under ambient light. These ROS species can oxidatively damage critical biomolecules, including membrane lipids, proteins, and nucleic acids, leading to compromised membrane integrity, leakage of cellular contents, and eventual bacterial death. In addition, direct physical contact between nanoparticles and the peptidoglycan layer may cause localized mechanical disruption, further accelerating cell lysis.

**Figure 9:**
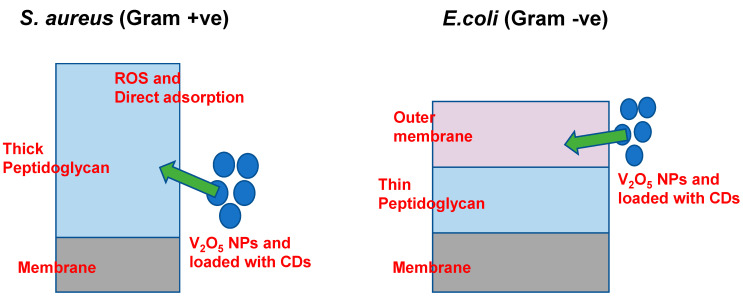
Mechanism of antimicrobial pathways.

In contrast, Gram-negative bacteria (*E. coli*) possess a more complex envelope architecture consisting of a thin peptidoglycan layer (~2–7 nm) sandwiched between the cytoplasmic membrane and an outer membrane rich in LPS. This outer membrane serves as a robust permeability barrier, limiting the diffusion and direct attachment of V_2_O_5_ NPs to the underlying peptidoglycan (Figure 9). Furthermore, the negatively charged LPS layer can cause electrostatic repulsion, reducing nanoparticle adsorption efficiency. Even in cases where some nanoparticles manage to interact with the outer membrane, the barrier properties impede ROS penetration to the cytoplasmic membrane, thereby attenuating antimicrobial efficacy. This explains the observed resistance of Gram-negative strains in agar well diffusion assays.

### 2.4. Limitations of the Current Study

While these observations provide preliminary evidence for the selective antimicrobial properties of V_2_O_5_-based NPs, the current study does not explore the detailed mechanistic pathways underlying these effects. A more comprehensive evaluation, examining NP cell surface interactions, ROS quantification, membrane integrity assays, and intracellular responses, is necessary to fully elucidate the mode of action. Such investigations will be the focus of our forthcoming research. The present results, however, clearly establish that CD-functionalized V_2_O_5_ NPs, especially MCD, exhibit enhanced antifungal and Gram-positive antibacterial activities, potentially expanding their applicability in targeted antimicrobial formulations.

### 2.5. Future Scope of the Current Study

The present findings demonstrate that CD-functionalized V_2_O_5_ NPs exhibit stable supramolecular assemblies with improved physicochemical properties, uniform distribution, and enhanced antimicrobial activity, particularly against Gram-positive bacteria and fungi. These attributes make them highly relevant for food packaging applications, where microbial contamination and spoilage are the major concerns. The V_2_O_5_:CD nanostructure provides a high surface area and diffusion channels, while BCD-based complexes regulate migration, enabling sustained and controlled release of antimicrobial agents when embedded in polymer-based food packaging films. The inclusion complexation confirmed by NMR suggests that the nanostructures can maintain stability in various environments, an essential feature for food-contact materials. Moreover, the synergistic antimicrobial action of V_2_O_5_ and CDs could provide an effective barrier against foodborne pathogens, potentially extending shelf life and reducing dependence on synthetic chemical preservatives. The incorporation of such nanostructures into biodegradable or polymer-based packaging films may also support the development of sustainable, multifunctional food packaging systems with both protective and eco-friendly characteristics.

## 3. Materials and Methods

### 3.1. Materials

Ammonium metavanadate (NH_4_VO_3_, ≥99% purity), ethanol (C_2_H_5_OH, ≥99.5%, analytical grade), and urea (CO(NH_2_)_2_, ≥99%) were procured from Sigma–Aldrich (St. Louis, MO, USA). Hydrochloric acid (HCl, 37% *w*/*w*) was purchased from Daejung Chemicals & Metals Co., Ltd. (Siheung-si, Republic of Korea). β-Cyclodextrin (BCD, ≥98%), hydroxypropyl-β-cyclodextrin (HCD, ≥98%), and methyl-β-cyclodextrin (MCD, ≥98%) were obtained from Tokyo Chemical Industry (TCI, Tokyo, Japan). All reagents were used without any further purification. Deionized (DI) water with a resistivity of 18.2 MΩ·cm, obtained from a Milli-Q purification system (Millipore, Burlington, MA, USA), was used throughout the experiments.

### 3.2. Synthesis of V_2_O_5_ NPs

A 0.1 M solution of ammonium metavanadate was prepared by dissolving the compound in 50 mL of hot distilled water, followed by the addition of 100 mL of ethanol. The pH of the resulting solution was adjusted to 3 using 50% diluted HCl. Upon acidification, the solution transformed into a colloidal, orange-colored dispersion. Subsequently, 0.5 g of urea was introduced into the mixture, which was then maintained at 50 °C under constant stirring for 90 min. A dark-brown precipitate formed during this process. The precipitate was collected, thoroughly washed, and centrifuged multiple times with distilled water and ethanol to remove any impurities. After washing, the sample was dried at 80 °C overnight and subsequently calcined at 550 °C for 2 h in a muffle furnace. The final product was a fenugreek yellow-colored V_2_O_5_ powder.

### 3.3. Synthesis of V_2_O_5_:BCD, V_2_O_5_:HCD, and V_2_O_5_:MCD

**Figure 10:**
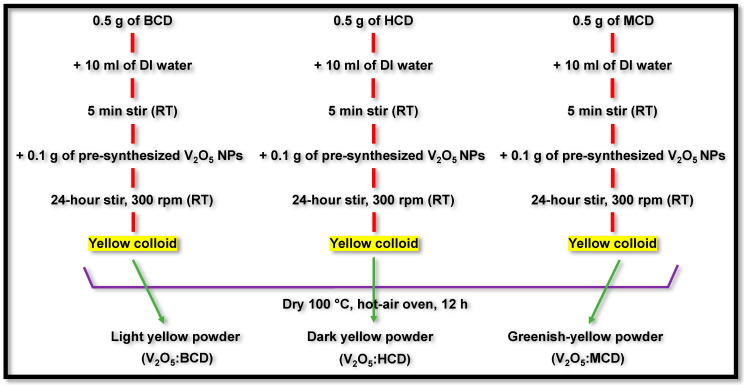
Schematic representation of the synthesis of V_2_O_5_:BCD, V_2_O_5_:HCD, and V_2_O_5_:MCD.

A total of 0.5 g each of BCD, HCD, and MCD was individually dissolved in 10 mL of distilled water. After stirring for 5 min, 0.1 g of the previously synthesized V_2_O_5_ was added to each solution. The mixtures were stirred at room temperature for 24 h at a constant speed of 300 rpm to ensure uniform dispersion of the V_2_O_5_ NPs within the CD solutions (Figure 10). Following the stirring period, the resulting yellow, colloidal solutions were dried at 100 °C for 12 h in a hot-air oven [28]. The final products obtained were light-yellow (V_2_O_5_:BCD), dark-yellow (V_2_O_5_:HCD), and greenish-yellow (V_2_O_5_:MCD) powders.

### 3.4. Antibacterial and Antifungal Activity (Agar Well Diffusion Method)

The *E. coli*, as a Gram-negative bacterium [ATCC 43895]; *S. aureus*, as a Gram-positive bacterium [ATCC 6538]; and the fungal *C. albicans* strain DAY185 were obtained from the Korean Culture Center of Microorganisms (KCCM, Seoul, Republic of Korea; http://www.kccm.or.kr accessed on 25 Aug 2017). Both bacterial strains were maintained and sub-cultured on sterile Mueller–Hinton agar (MHA) or Mueller–Hinton broth (MHB), and the fungal strain *C. albicans DAY185* was maintained and sub-cultured on Potato Dextrose Agar (PDA) or in Potato Dextrose Broth (PDB). To check the antimicrobial efficacy of the prepared nanohybrid, as previously reported [42,43], samples were prepared by dissolving them in 100% DMSO. MHA and/or PDA plates were uniformly swabbed with 100 µL of the respective bacterial (~107 CFU/mL) or fungal (~10^5^ CFU/mL) suspension. Agar plates were pierced with a 9 mm-diameter cork borer and loaded with 100 μL of V_2_O_5_, V_2_O_5_:BCD, V_2_O_5_:HCD, and V_2_O_5_:MCD, and then incubated for 24 h, or, in the case of *C. albicans*, for 48 h, at 37 °C. After incubation, the diameter of the clear inhibition zone surrounding each well was measured in millimeters using a digital Vernier caliper. The reported values represent the mean inhibition zone diameter excluding the initial 9 mm well size. Clinical Laboratory Standards Institute (CLSI) guidelines for bacteria (CLSI M100, 2015 and Clinical Laboratory Standards Institute (CLSI) guidelines for yeasts (CLSI M44, 2017) were applied. Antimicrobial analysis of each sample was conducted in duplicate, and the average of zones of inhibition were calculated and recorded. All experiments were performed using at least two independent biological cultures, with each assay conducted in triplicate.

### 3.5. Instruments Used

The material was comprehensively characterized using ^1^H NMR, FE-SEM, and powder XRD techniques. The ^1^H NMR spectrum was recorded on a Bruker 600 MHz spectrometer. FE-SEM analysis was carried out on a Hitachi S-4800 instrument equipped with EDX, operated at an accelerating voltage of ~10 kV. For FE-SEM, samples were mounted on double-sided carbon tape without any additional coating, and images were captured at multiple magnifications. Powder XRD measurements were performed on a PANalytical X’Pert^3^ MRD diffractometer using monochromatized Cu K_α_ radiation (λ = 1.54 Å) at 30 mA and 40 kV. All instrumental facilities were utilized at the Core Research Support Center for Natural Products and Medical Materials, Yeungnam University.

## 4. Conclusions

This study reports the synthesis of V_2_O_5_ NPs integrated with BCD and its derivatives HCD and MCD, using precipitation and co-precipitation methods. The formation of the V_2_O_5_:CD supramolecular assemblies was achieved through non-covalent host–guest interactions, resulting in stable NPs with improved physicochemical properties. SEM analysis revealed a porous, flower-like morphology with uniform V_2_O_5_ NP distribution within the CD. Proton NMR spectroscopy confirmed the inclusion complexation between V_2_O_5_ and the CDs. The NPs exhibited enhanced antimicrobial performance, particularly against Gram-positive bacterial and fungal strains, attributed to the synergistic effects of CD-assisted stabilization, dispersion, and controlled release. These findings suggest that CD-functionalized V_2_O_5_ NPs hold strong potential as multifunctional antimicrobial agents for biomedical and environmental applications.

## Figures and Tables

**Table 1:** Antibacterial and antifungal efficacy of V_2_O_5_, V_2_O_5_:BCD, V_2_O_5_:HCD, and V_2_O_5_:MCD against *S. aureus*, *E. coli*, and *C. albicans* by zone of inhibition (values exclude the 9 mm well diameter). Data are presented as mean ± SD from two independent biological experiments, each performed in triplicate.

Bacterial Strains	Zone of Inhibition (mm)			
	Control (V_2_O_5_)	(V_2_O_5_:BCD)	(V_2_O_5_:HCD)	(V_2_O_5_:MCD)
*S. aureus*	7.3 ± 0.2	8.5 ± 0.3	9.3 ± 0.9	11.4 ± 0.9
*E. coli*	0.0 ± 0.0	0.0 ± 0.0	0.0 ± 0.0	0.0 ± 0.0
*C. albicans*	6.2 ± 0.3	6.4 ± 0.1	7.8 ± 0.4	10.2 ± 0.2

## Data Availability

The datasets used or analyzed during the current study are available from the corresponding author upon reasonable request.

## Supplementary Materials

The following supporting information can be downloaded at: https://www.mdpi.com/article/10.3390/bioengineering12101010/s1, Figure S1. ^1^H NMR spectra of BCD; Figure S2. ^1^H NMR spectra of HCD; Figure S3. ^1^H NMR spectra of MCD; Figure S4. Zones of inhibition of prepared materials with *C. albicans, S. aureus*, and *E. coli*; Figure S5. Antibacterial activity of V_2_O_5_, V_2_O_5_:BCD, V_2_O_5_:HCD, V_2_O_5_:MCD against and *E. coli* ATCC 43895; Table S1. Proton Chemical Shifts of BCD, and V_2_O_5_:BCD; Table S2. Proton Chemical Shifts of HCD and V_2_O_5_:HCD; Table S3. Proton Chemical Shifts of MCD, and V_2_O_5_:MCD.
