# Supplementary material for: Synthesis of the Supramolecular Structure of Vanadium Pentoxide Nanoparticles with Native and Modified β-Cyclodextrins for Antimicrobial Performance
Source: Bioengineering (Basel). 2025 Sep 23;12(10):1010. doi: 10.3390/bioengineering12101010 (PMC12561820; doi:10.3390/bioengineering12101010)
Supplement: Supplementary file 1 [file bioengineering-12-01010-s001.zip › bioengineering-3856691-supplementary.pdf]

Synthesis of the supramolecular structure of vanadium pentoxide nanoparticles with β-Cyclodextrin for anti-microbial performances

Rajaram Rajamohan[a*, 1], Kanagaraj Thamaraiselvi[a, 1], Chaitany Jayprakash Raorane[a], Kuppusamy Murugavel[b], Chandramohan Govindasamy[c], Seong-Cheol Kim[a], Seho Sun[a*]

[a] School of Chemical Engineering, Yeungnam University, Gyeongsan 38541, Republic of Korea.

[b] PG and Research Department of Chemistry, Government Arts College, Chidambaram - 608 102, Tamil Nadu, India.

[c] Department of Community Health Sciences, College of Applied Medical Sciences, King Saud University, P.O. Box 10219, Riyadh 11433, Saudi Arabia.

*Correspondence: rajmohanau@yu.ac.kr (R.R); seho.sun@yu.ac.kr (S.S)

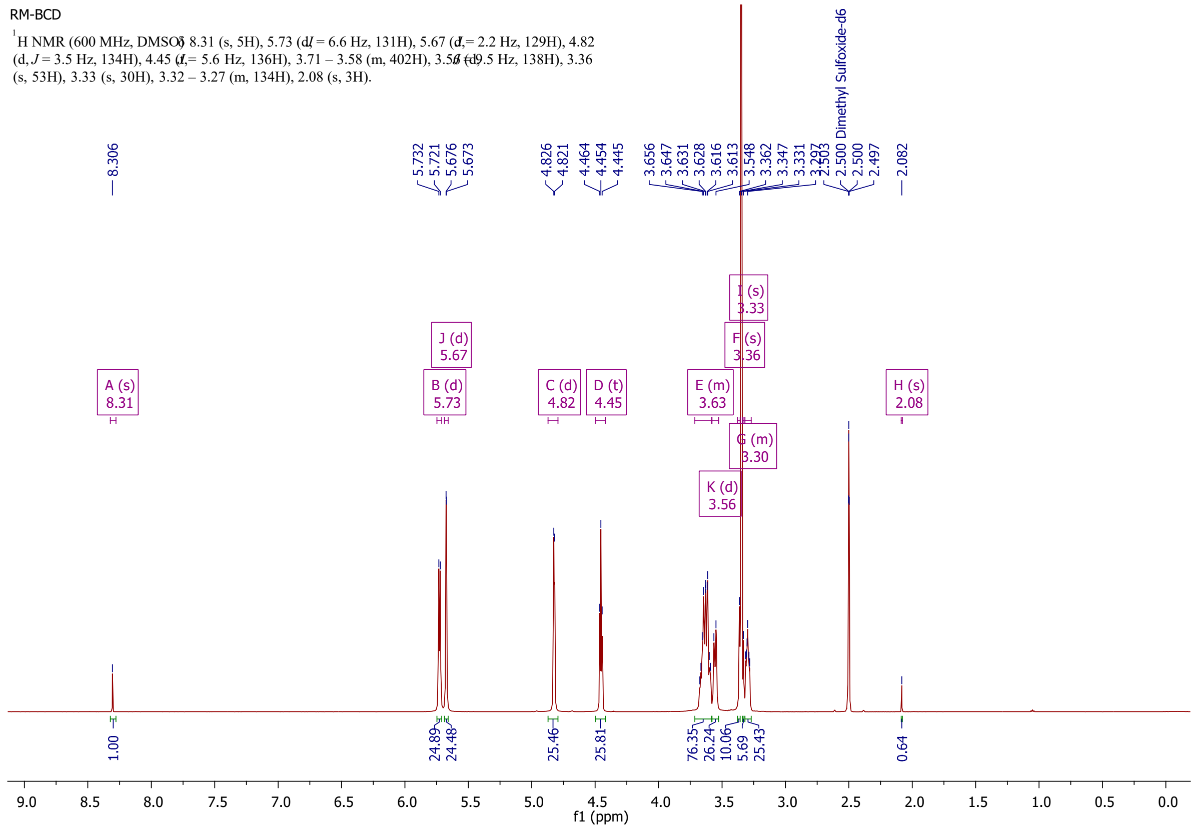

Figure S1. ^{1}H NMR spectra of BCD

Table S1. Proton Chemical Shifts of BCD, and V_2O_5:BCD

Positions	Chemical shifts (ppm)	
	**BCD**	**V_2O_5:BCD**
H1	4.82 (d, 3.6 Hz)	4.82 (d, 6 Hz)
H2	3.28-3.31 (m)	3.3 (multiplet merged with dmso-d6 water peak
H3	3.63 (m, merged with CH2 protons)	3.60-3.66 (m)
H4	3.56 (d, 9.6 Hz)	3.55 d (18 Hz)
H5	3.59-3.68 (m)	3.60-3.66 (m)
CH_2 Protons	3.59-3.68 (m)	3.60-3.66 (m)
Equatorial OH protons at C-2	5.73 (d, 7.2 Hz)	5.72 (d, 6 Hz)
Equatorial OH protons at C-3	5.68 (d, 3 Hz)	5.67 (d, 6 Hz)
OH of CH_2 at C-5	4.46 (t, 5.4 Hz)	4.45 (t)

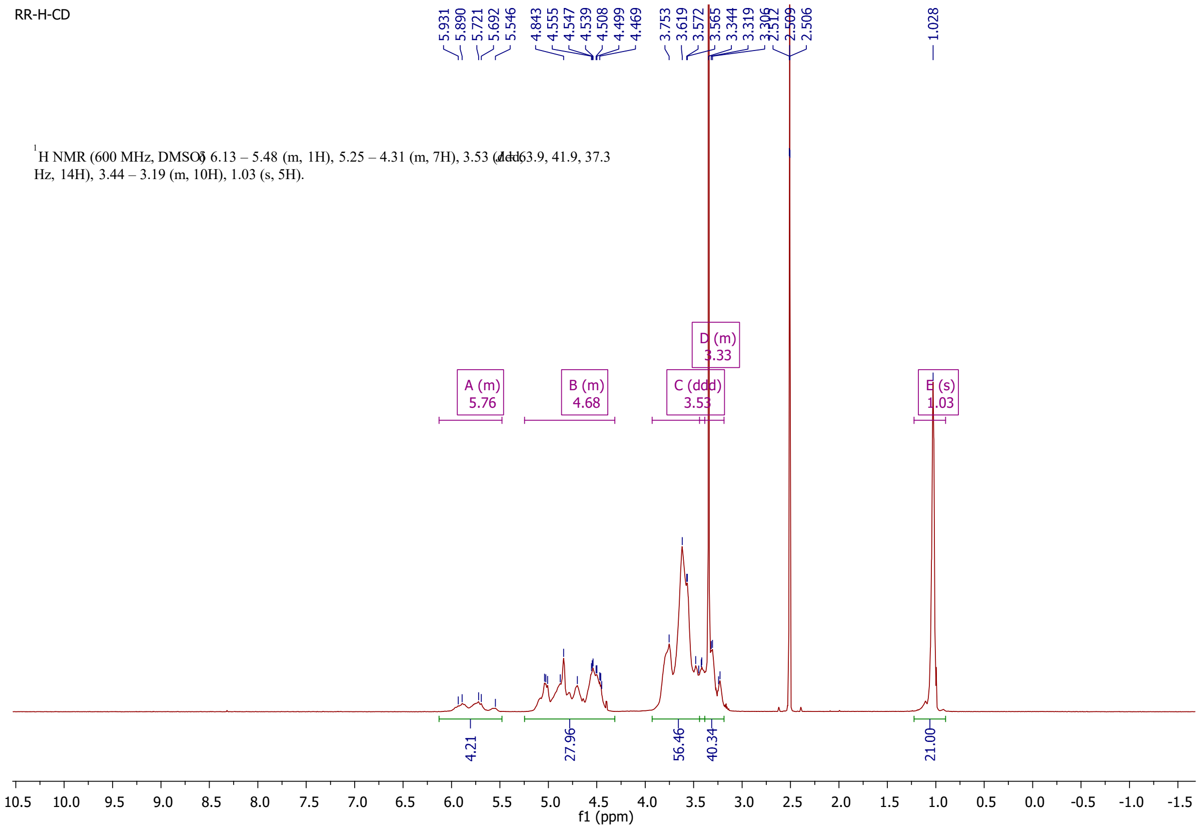

Figure S2. ^{1}H NMR spectra of HCD

Table S2. Proton Chemical Shifts of HCD and V_2O_5:HCD

Positions	Chemical shifts (ppm)	
	**HCD**	**V_2O_5:HCD**
H1 (Equatorial hydrogen)	4.84 (m)	5.00-5.04 (m)
H2	3.30-3.31 (m)	3.30-3.31 (m)
H3	3.56-3.75 (m)	3.55-3.75 (m)
H4	3.56-3.75 (m)	3.55-3.75 (m)
H5	3.56-3.75 (m)	3.55-3.75 (m)
CH_2 protons at C-5	3.56-3.75 (m)	3.55-3.75 (m)
Equatorial OH protons at C-2	4.53-4.55 (m)	4.83-4.87 (m)
Equatorial OH protons at C-3	4.53-4.55 (m)	4.83-4.87 (m)
The methyl proton of the propyl group	1.03 (m)	1.01-1.03 (m)
The methylene proton of the propyl group	3.56-3.75 (m)	3.55-3.75 (m)
Methine proton of the propyl group	4.46-4.50 (m)	4.53-4.77 (m)
The hydroxy proton is attached to the propyl groups	5.54-5.93 (m)	5.55-5.88 (m)

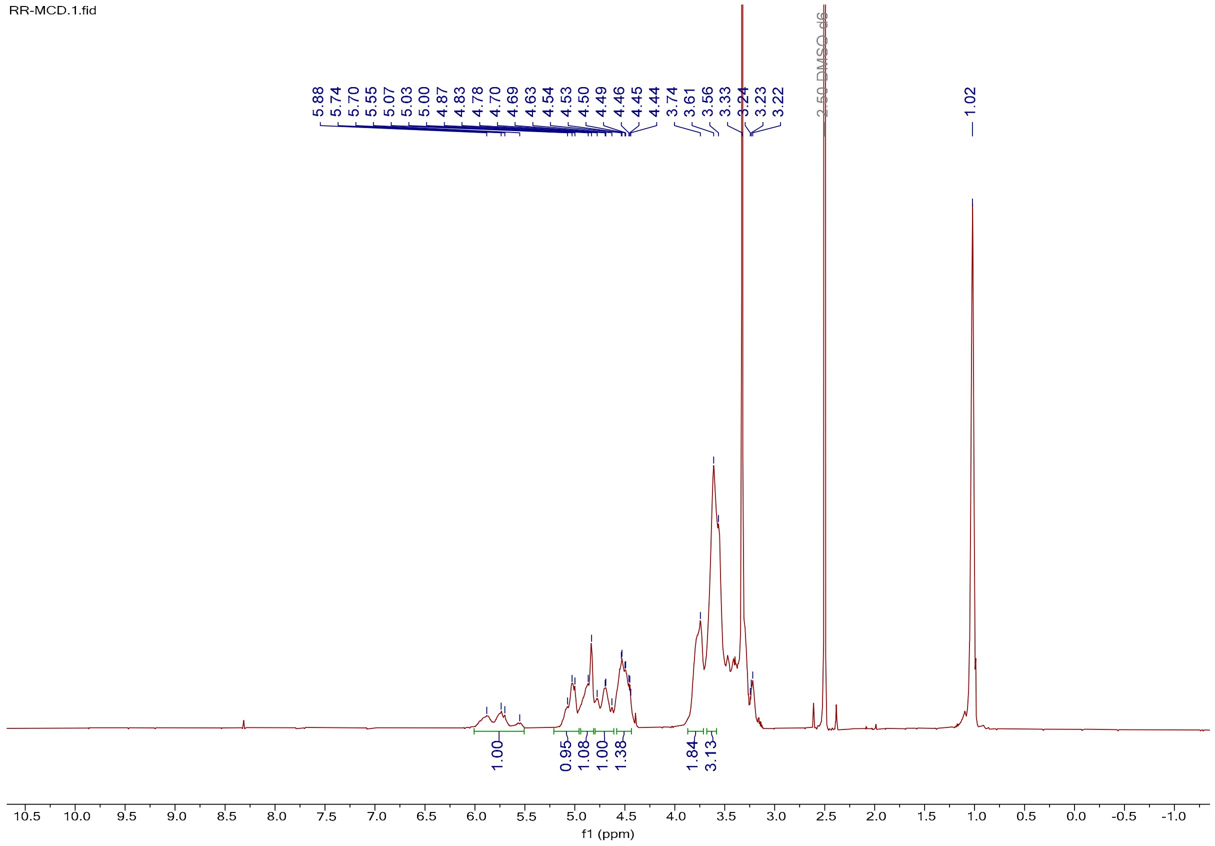

Figure S3. ^{1}H NMR spectra of MCD

Table S3. Proton Chemical Shifts of MCD, and V_2O_5:MCD

**Positions**	**Chemical shifts (ppm)**	
	**MCD**	**V_2O_5:MCD**
H1	5,74, m	5.10, bs
H2	3.23 (m)	3.38 (m)
H3	4.72-5.03 (m) merged	4.53 (m)
H4	4.72-5.03 (m) merged	4.76-4.93 m
H5	4.72-5.03 (m) merged	5.03 (m)
CH_2 Protons	4,45-4.54 (m)	3.49 & 3.70 (m)
Equatorial OMe protons at C-2	3.56 (s)	3.25 bs
Equatorial OMe protons at C-3	3.74 (s)	3.58 (merged)
OMe of CH_2 at C-5	3.61 (s)	3.50 bs

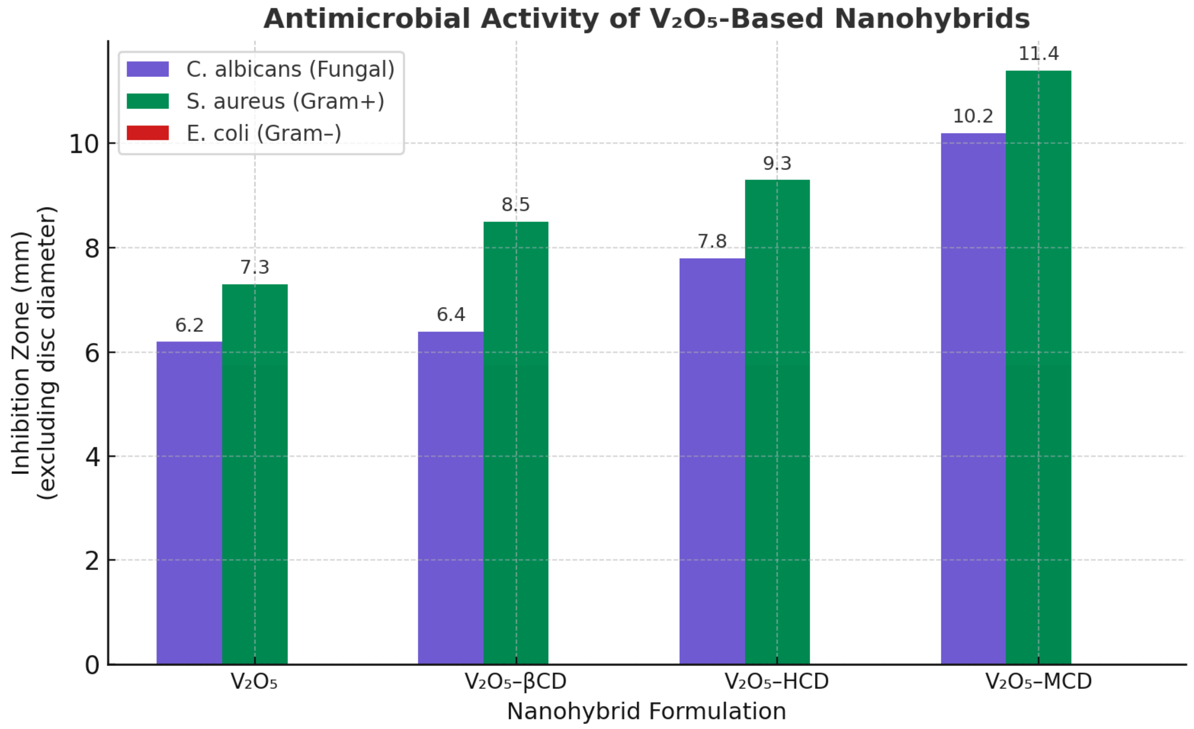

Figure S4. Zones of inhibition of prepared materials with *C. albicans*, *S. aureus*, and *E. coli.*

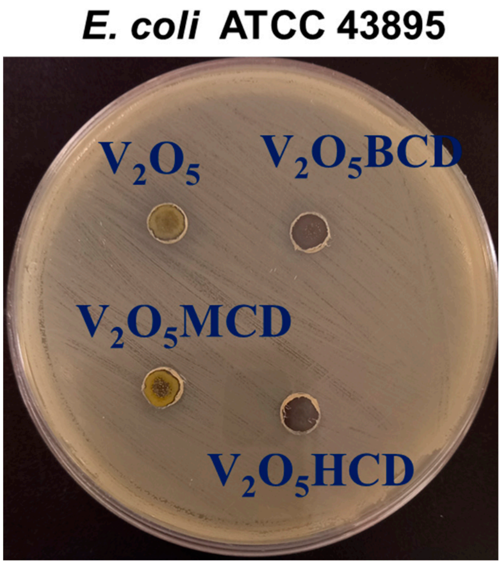

Figure S5. Antibacterial activity of V_2O_5, V_2O_5:BCD, V_2O_5:HCD, V_2O_5:MCD against and *E. coli* ATCC 43895.
